# Metabolic traits of an uncultured archaeal lineage -MSBL1- from brine pools of the Red Sea

**DOI:** 10.1038/srep19181

**Published:** 2016-01-13

**Authors:** Romano Mwirichia, Intikhab Alam, Mamoon Rashid, Manikandan Vinu, Wail Ba-Alawi, Allan Anthony Kamau, David Kamanda Ngugi, Markus Göker, Hans-Peter Klenk, Vladimir Bajic, Ulrich Stingl

**Affiliations:** 1 Red Sea Research Center, King Abdullah University of Science and Technology (KAUST), Thuwal, Saudi Arabia; 2 Computational Bioscience Research Center, King Abdullah University of Science and Technology (KAUST), Thuwal, Saudi Arabia; 3 German Collection for Microorganisms and Cell Cultures GmbH (DSMZ), Inhoffenstraße 7b, 38124 Braunschweig, Germany; 4 School of Biology, Newcastle University, Newcastle upon Tyne, United Kingdom

## Abstract

The candidate Division MSBL1 (**M**editerranean **S**ea **B**rine **L**akes 1) comprises a monophyletic group of uncultured archaea found in different hypersaline environments. Previous studies propose methanogenesis as the main metabolism. Here, we describe a metabolic reconstruction of MSBL1 based on 32 single-cell amplified genomes from Brine Pools of the Red Sea (Atlantis II, Discovery, Nereus, Erba and Kebrit). Phylogeny based on rRNA genes as well as conserved single copy genes delineates the group as a putative novel lineage of archaea. Our analysis shows that MSBL1 may ferment glucose via the Embden–Meyerhof–Parnas pathway. However, in the absence of organic carbon, carbon dioxide may be fixed via the ribulose bisphosphate carboxylase, Wood-Ljungdahl pathway or reductive TCA cycle. Therefore, based on the occurrence of genes for glycolysis, absence of the core genes found in genomes of all sequenced methanogens and the phylogenetic position, we hypothesize that the MSBL1 are not methanogens, but probably sugar-fermenting organisms capable of autotrophic growth. Such a mixotrophic lifestyle would confer survival advantage (or possibly provide a unique narrow niche) when glucose and other fermentable sugars are not available.

More than half of the 60 major lines of descent within the bacterial and archaeal domains that have been described based on SSU rRNA phylogeny^1^ remain uncultured and make up the so-called “microbial dark matter”^2^, since their metabolic capabilities and ecological role remain obscure. Members of the candidate division MSBL1 (**M**editerranean **S**ea **B**rine **L**akes 1) encompass an uncultured archaeal lineage that is abundant and widespread in deep hyper-saline anoxic basins (DHABs) of the Mediterranean Sea, the Red Sea, and the Gulf of Mexico^3^,^4^,^5^. 16S rRNA signature sequences of this group were also reported from the anoxic hypolimnion of a shallow hyper-saline Solar Lake in Egypt^6^, sediments of hyper-saline Lake Chaka in China^7^, from a crystallizer in a multi-pond solar saltern in the south of Mallorca Island^8^,^9^ and recently in metagenomic libraries from a hyper-saline lake in Kenya (Mwirichia *et al*. unpublished data). MSBL1 have been postulated to be methanogenic based on their phylogenetic position and circumstantially because they are the numerically dominant archaeal group in DHABs, where incidentally also high methane concentrations of presumably biogenic origin are present^3^,^10^,^4^,^11^. However, with the exception of few sequences distantly related to *Methanohalophilus*^4^,^12^ no other homologs of the key methanogenic enzyme, methyl coenzyme-M reductase (*mcr*A) have been recovered from the brine pools studied so far. Therefore, the exact metabolism of this group remains enigmatic in the absence of cultured representatives or larger contigs of genomic sequences. In this study, we applied single-cell genomics using cells of the MSBL1 clade from the Red Sea brine pools to reconstruct their metabolic potential. Our study provides the first evidence of their non-methanogenic metabolic capabilities that enable them to thrive in the anoxic DHABs.

## Results

### Genome analysis

**Figure 1:**
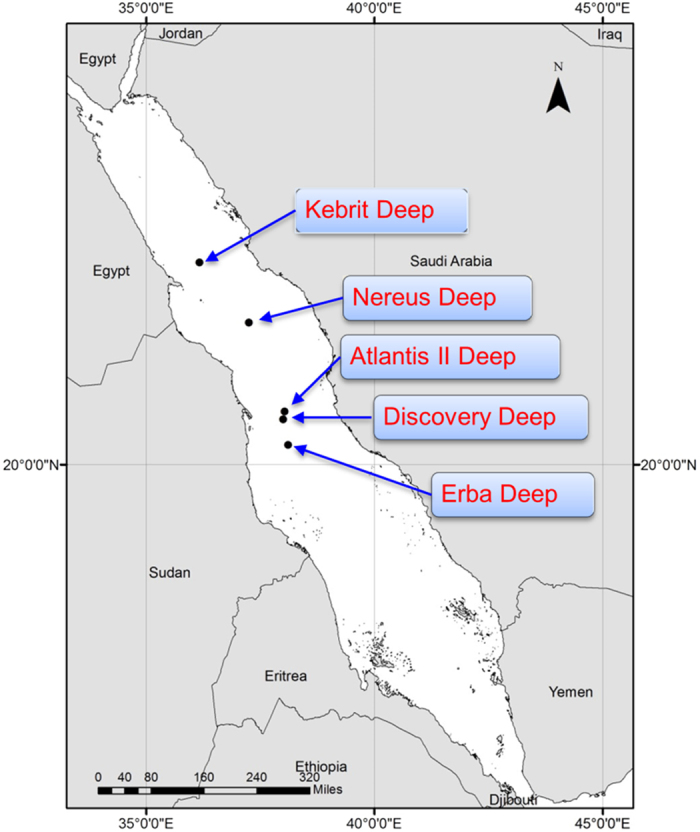
Map of the Red Sea, showing five different deep-sea brines along the N-S axis of the Red Sea. The map was generated using ArcGIS v.10.1.

The physico-chemistries of the different Red Sea brines have been described previously^5^,^13^. A total of thirty-two single-cell amplified genomes (SAGs) described in this study were recovered from four brine pools (Atlantis II, Discovery, Erba, and Nereus) in the Red Sea (Fig. 1).

The origins of the single cell genomes are as follows-Atlantis II (8), Discovery (13), Erba (3) and Nereus Deep (9). The features of the assembled single cell genomes including size (137,797 – 1,424,127 bases) and estimated genome completeness (0.0% – 56.7%), as evaluated using a set of 104 conserved single-copy archaeal genes as described by AMPHORA2^14^ are shown in Table 1.

**Figure 2:**
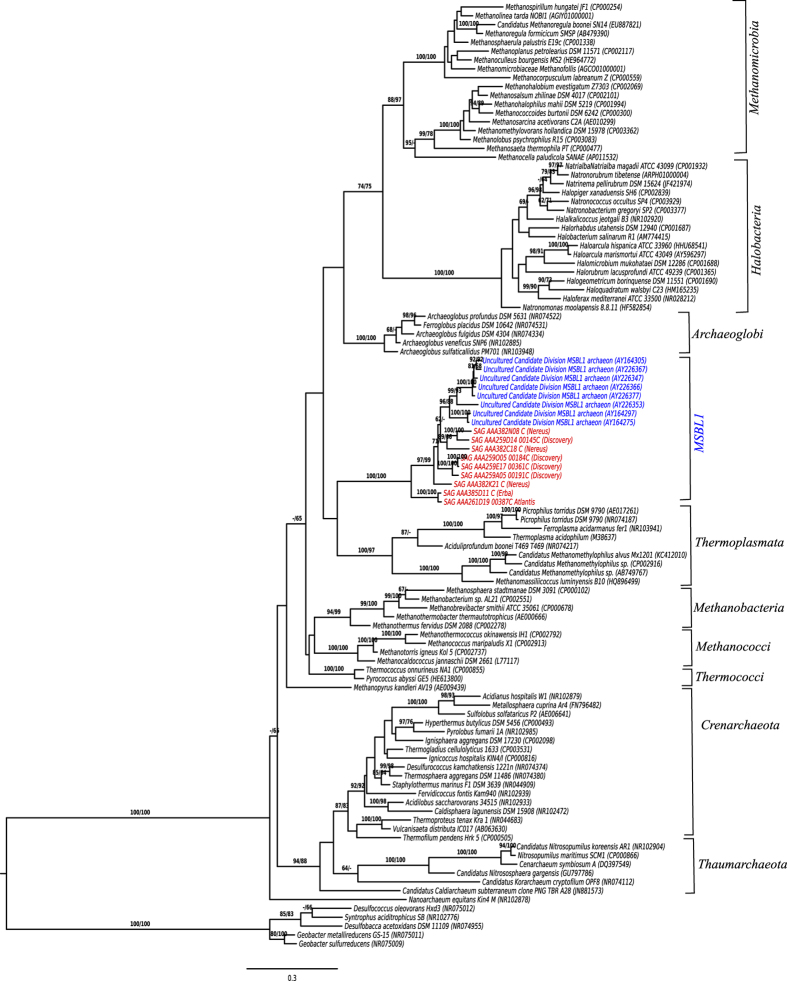
Maximum likelihood (ML) phylogenetic tree inferred using only nearly complete 16S rRNA genes from SAGs of Brine Pools from the Red Sea (in red) and comparative sequences from the NCBI database including MSBL1 sequences from Brine Pools in the Mediterranean (blue). The branches are scaled in terms of the expected number of substitutions per site. Numbers above branches are support values from ML (left) and maximum parsimony (MP; right) bootstrapping. The tree was rooted with selected bacterial sequences.

**Figure 3:**
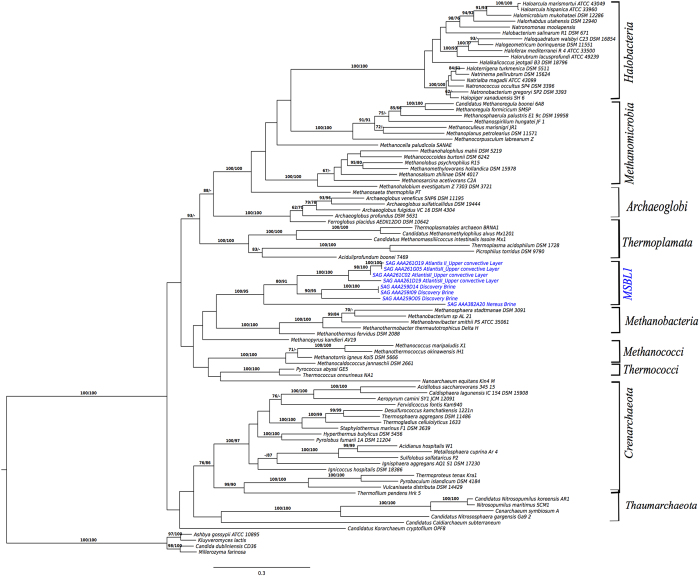
Maximum likelihood (ML) phylogenetic tree inferred from the amino-acid matrix of 10 concatenated archaeal proteins present in eight of the MSBL1 SAGs (in blue) and other archaeal genomes. The same set of proteins from selected Eukaryota was included as out-group. The branches are scaled in terms of the expected number of substitutions per site. Numbers above branches are bootstrapping support values from ML (left) and maximum parsimony (MP; right). The ‘prot’ amino-acid matrix comprised 94 operational taxonomic units and 1305 characters, 1207 of which were variable and 1159 of which were parsimony-informative. ML analysis under the LG model yielded a highest log likelihood of -104791.64, whereas the estimated alpha parameter was 0.95. The bootstrapping converged after 350 replicates; the average support was 78.52%. MP analysis yielded a best score of 20784 (consistency index 0.32, retention index 0.59) and 14 best tree(s). The MP bootstrapping average support was 73.25%.

Genome completeness computed using a larger set of 191 genes described by Dodsworth *et al*.^15^ shows that best result/completeness was 54.2% in the SAG AAA259I09, a slight variation from 56.7% shown in AMPHORA^14^. The complete set of 191 genes (including those described in AMPHORA) is listed in Supplementary Table S1. Phylogenetic inference using the full-length 16S rRNA gene sequences^16^ delineates the MSBL1 as a novel deep-branching order, distinct from the described methanogens (Fig. 2). The matrix for the 16S rRNA gene sequences comprised 107 operational taxonomic units and 1714 characters, 1269 of which were variable and 1070 of which were parsimony-informative. ML analysis under the GTR model yielded a highest log likelihood of -47688.24, whereas the estimated alpha parameter was 0.52. The bootstrapping converged after 650 replicates; the average support was 74.73%. MP analysis yielded a best score of 10431 (consistency index 0.24, retention index 0.69) and 2 best tree(s). The MP bootstrapping average support was 70.88%. On the basis of the Silva aligner^17^, members of the MSBL1 are placed within the class *Thermoplasmata* with identity scores between 83 to 86.9%. A phylogenetic tree based on partial 16S rRNA genes and encompassing MSBL1 sequences from other environments is shown in Supplementary Figure S1. In this tree, the sequences from the Red Sea brine pools cluster with those from brine pools in the Mediterranean Sea. Complementary phylogenetic analyses using a concatenated set of ten conserved single-copy genes present in 8 MSBL1 SAGs and other sequenced archaeal genomes (Fig. 3) also confirms the placement of MSBL1 as a novel archaeal lineage distinct from methanogens. Unexpectedly, we found that, although this novel archaeal group preferentially occurs in hyper saline environments, the largely unimodal distribution in the isoelectric point (*p*I) of their overall protein-coding genes places their proteomes in the same range as moderate halophiles (Supplementary Figure S2). An exception is the genome AAA385M11, whose *p*I is slightly higher, which may be an artefact due to the small size (215 Kb) of the assembled genome.

### Carbohydrate transport and metabolism

Genes encoding for transcriptional regulators involved in carbohydrate transport and metabolism were identified in 18 of the SAGs (Supplementary Table S2), the most important being the archaeal sugar-specific transcriptional regulator *Trm*B. In *Thermococcus litoralis*, the respective protein is involved in the maltose-specific regulation of a gene cluster (*mal*E, *mal*F, *mal*G, *mal*K) that encodes a trehalose/maltose-binding protein-dependent ABC transporter for trehalose and maltose^18^. The genes *malE* and *malG* were identified in the genomes of AAA259A05 and AAA259E19, respectively. MalE is a maltose binding protein whereas MalG is a maltose transport system permease. Sugar transporters include a putative catabolism phosphotransferase system, putative sugar ABC transport system and a glucose import ATP-binding protein TsgD13 (Supplementary Table S2). Potential substrates include glucose, galactose arabinose, maltodextrin, maltose, xylose and ribose (Supplementary Table S2). Trehalose could play a significant role both as a carbon source and also compatible solute involved in osmoprotection. In this group, trehalose is synthesized from maltose, starch or UDP glucose (Supplementary Table S2; Supplementary Figure S3). The ability to utilize trehalose as an osmolyte would explain their rather normal pI as compared to that of other extreme halophiles. In the genome of AAA259B11, α-D-glucanotransferase may be involved in conversion of starch to trehalose. Supplementary Figure S3 summarizes the initial sugar metabolism to either α-D-glucose or trehalose.

### Glycolysis/ Gluconeogenesis

**Figure 4:**
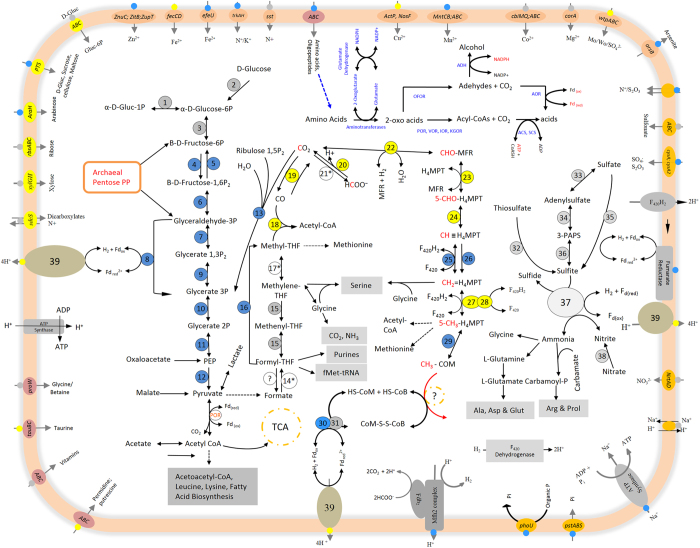
A putative global metabolism of the MSBL1 based on 32 Single Amplified Genomes. The figure summarizes glycolysis/gluconeogenesis, autotrophic carbon fixation, one-carbon metabolism via the tetrahydrofolate/tetrahydromethanopterin pathways, sulfur, nitrogen, amino acid degradation and aldehyde metabolism. Membrane associated proteins, proteins involved in solute or ion transport are anchored in the membrane and the arrows indicate the flow direction (import, export or symport). Encircled numbers represent the various enzymes, whereas the color of the tiny balls on the periphery indicate in how many of the SAGs was the enzyme identified: Grey color 1–5 SAGs, Blue 6–10; Yellow 11–16 SAGs. * denotes not detected. The enzymes are: (**1**) phosphoglucomutase; (**2**) PTS system cellobiose-specific IIA component protein; (**3**) glucose-6-phosphate isomerase; (**4**) 6-phosphofructokinase/Pyrophosphate—fructose 6-phosphate 1-phosphotransferase protein; (**5**) fructose 16-bisphosphate aldolase; (**6**) fructose 16-bisphosphate aldolase-phosphatase protein; (**7**) glyceraldehyde-3-phosphate dehydrogenase; (**8**) tungsten-containing aldehyde ferredoxin oxidoreductase (GAPOR)/Aldehyde oxidoreductase protein; (**9**) phosphoglycerate kinase protein; (**10**) 2,3-bisphosphoglycerate-dependent phosphoglycerate mutase/2,3-bisphosphoglycerate-dependent phosphoglycerate mutase; (**11**) enolase; (**12**) pyruvate kinase protein; **(13)** ribulose bisphosphate carboxylase protein; (14*) Formate-tetrahydrofolate ligase is missing; (**15**) bifunctional protein FolD; (**16**) putative thymidylate synthase protein/5-methyltetrahydrofolate-homocysteine methyltransferase; (17*) methylenetetrahydrofolate reductase; (**18**) acetyl-CoA synthase (+Ni, Fe), carbon monoxide dehydrogenase; corrinoid protein (**19**) Acetyl-CoA decarbonylase-synthase complex/Carbon monoxide dehydrogenase; (**20**) formate dehydrogenase; (21*) tungsten-containing hydrogen dependent formate dehydrogenase); (**22**) formylmethanofuran dehydrogenase; (**23**) formylmethanofuran-tetrahydromethanopterin formyltransferase; (**24**) methenyltetrahydromethanopterin cyclohydrolase; (**25**) coenzyme F_420_-dependent N-methenyltetrahydromethanopterin dehydrogenase; (**26**) methylene-tetrahydromethanopterin dehydrogenase; (**27**) 5,10-methylenetetrahydromethanopterin reductase; (**28**) coenzyme F_420_ hydrogenase; (**29**) tetrahydromethanopterin S-methyltransferase; (**30**) CoB—CoM heterodisulfide reductase; (3**1**) coenzyme F_420_-reducing hydrogenase; (**32**) thiosulfate sulfurtransferase GlpE protein; (**33**) sulfate adenylyltransferase protein; (**34**) adenylylsulfate kinase protein/ Probable adenylyl-sulfate kinase protein; (**35**) sulfoxide reductase catalytic subunit YedY protein; (**36**) sulfite oxidase protein/ phosphoadenosine phosphosulfate reductase protein; (**37**) ferredoxin-nitrite reductase protein/ sulfite reductase ferredoxin 2 protein; (**38**) periplasmic nitrate reductase protein; (**39**) NADH-quinone oxidoreductase. *Formate—tetrahydrofolate ligase is missing. Enzymes involved in amino acid degradation are labelled as: (**ADH**) Alcohol dehydrogenase; (**OFOR**) 2-Oxoacid:ferredoxin oxidoreductase; (**AOR**) tungsten-containing aldehyde ferredoxin oxidoreductase; (**POR**) Pyruvate ferredoxin oxidoreductase; (**VOR**) 2-ketoisovalerate ferredoxin oxidoreductase; (**IOR**) indolepyruvate: ferredoxin oxidoreductase; (**KGOR**) 2-Oxoglutarate ferredoxin oxidoreductase subunit beta; (**ACS**) acetyl-CoA synthetase II (NDP forming); (**SCS**) archaeal succinyl-CoA synthetase (NDP forming).

Diversity in sugar metabolism pathways in archaea as well as the variability in enzymes involved has been reviewed recently^19^. The MSBL1 group uses a fermentative sugar metabolism that combines the classical and recently discovered (archaeal) enzymes of the Embden-Meyerhof (EM) pathway (Fig. 4; Supplementary Table S2). The absence of cytochromes, cytochrome oxidases and quinones in all the SAGs reinforce our hypothesis that these Archaea are likely to ferment and also that they probably do not contain an electron transport chain. Besides, presence of oxygen-sensitive enzymes (pyruvate-ferredoxin oxidoreductase) and absence of catalase indicates a strictly anaerobic lifestyle as expected within the anoxic brine environment.

During sugar metabolism via the EM pathway, glucose is converted to two molecules of pyruvate and yields two ATPs, reducing equivalents and intermediates that are precursors for cellular building blocks. The products of sugar fermentation are acetate, carbon dioxide and H_2_. The two key genes for the alternative Entner-Doudoroff pathway, gluconate dehydratase and KDG aldolase are missing in all the SAGs, which could be related to the fact this pathway has one less ATP net yield compared to the EM pathway that yields two ATP molecules. As illustrated in Fig. 3, the transported glucose molecules or those emanating from the hydrolysis of cellulose are probably converted to α-D-glucose 1-phosphate and s α-D-glucose-6-phosphate, eventually entering the Embden-Meyerhof pathway. The genes involved in glycolysis are summarized in Supplementary Table S2. Archaeal glyceraldehyde-3-phosphate ferredoxin oxidoreductase was only identified in the genome of AAA259A05. Usually, the conversion of pyruvate to phosphoenolpyruvate is catalyzed by phosphoenolpyruvate synthase (EC 2.7.9.2). However, during gluconeogenesis, phosphoenolpyruvate can also be synthesized from oxaloacetate by phosphoenolpyruvate carboxykinase (EC 4.1.1.32) present in eleven of the SAGs. Similar to the haloarchaea, the MSBL1 group does not encode archaeal-type pyruvate carboxylase which catalyses the irreversible carboxylation of pyruvate to oxaloacetate^20^.

### Pentose metabolism

The oxidative pentose phosphate pathway is lacking in all the SAGs, consistent with findings in other archaea. Instead, pentoses are metabolized non-oxidatively by conversion of fructose 6-phosphate (C_6_) to ribulose 5-phosphate (C_5_). The four enzymes required in this archaeal pathway (fructose 1,6 bisphosphatase, fructose 16-bisphosphatase, 6-phospho-3-hexuloisomerase and 3-hexulose-6-phosphate synthase) were identified in ten of the genomes (Supplementary Table S2). Another source of the ribulose 5-phosphate could be ribose sugars via the nucleotide salvage pathway. Ribulose bisphosphate carboxylase (identified in 14 of the SAGs) is an enzyme known to convert ribulose 1,5-biphosphate to the highly unstable six-carbon intermediate 3-keto-2-carboxyarabinitol 1,5-bisphosphate, which spontaneously decays to two molecules of glycerate 3-phosphate. This end product is fed into the central metabolic pathway. The ribulose bisphosphate carboxylase proteins identified in nine of the SAGs are phylogenetically closely related to the archaeal form III cluster of RuBisCo proteins, which are able to fix CO_2_ to ribulose bisphosphate^21^. These form III RuBisCo proteins have also been shown to participate in the AMP salvage pathway^22^. In the genome of AAA259A05, Glyceladehyde-3P is synthesized from deoxyribose sugars catalysed by ribokinase/phosphopentomutase and deoxyribose-phosphate aldolase. The enzymes involved in the different reactions are listed in Supplementary Table S2.

### Carbon fixation

Though the genomes of the individual SAGs are largely incomplete, the complete TCA cycle was recovered in AAA259A05 and AAA259I09 and the genes involved are listed in Supplementary Table S2. MSBL1 possesses genes that are typically involved in autotrophic and anaplerotic CO_2_ fixation. The reductive TCA cycle leads to the fixation of two molecules of CO_2_ and the production of one molecule of acetyl-CoA catalysed by the key enzymes 2-oxoglutarate-ferredoxin oxidoreductase and isocitrate dehydrogenase. These two genes were identified in seven SAGs indicating that MSBL1 may have a functional reductive citric acid cycle. However, ATP-citrate lyase, which catalyses an ATP-dependent cleavage of citrate to oxaloacetate and acetyl-coA was not detected in any of the SAGs. Instead, two homologs were identified in the genomes AAA261O19 (gene.AAA261O19_00625C) and AAA261C02 (gene.AAA261C02_00763C) albeit with low similarity value of 32% to the known enzyme. In the anaplerotic reaction acetyl-CoA is (reversibly) reductively carboxylated to pyruvate by pyruvate:ferredoxin oxidoreductase (*porA, porB, porC* were identified in nine genomes) from which all other central metabolites can be formed or used for gluconeogenesis via a reversal of the EMP pathway. Alternatively, the enzyme phosphoenolpyruvate carboxylase (present in SAGs AAA382A03_00089C and AAA382N08) is able to fix CO_2_ by using phosphoenolpyruvate^23^. Neither pyruvate decarboxylase, which catalyses the decarboxylation of pyruvic acid to acetaldehyde and carbon dioxide, nor lactate dehydrogenase were detected in any of the SAGs. A glyoxylate bypass is also probably missing as the two key genes (isocitrate lyase and malate synthase) were not detected. However, the organisms may import and degrade a variety of organic acids since beta-oxidation enzymes such as ferredoxin-dependent oxidoreductases are present (Supplementary Table S2). Acquisition of amino acids and proteins from the surrounding environment is evidenced by the occurrence of binding/transport proteins for branched chain amino acids as well as oligopeptides (Supplementary Table S2). Beta-oxidation of the branched chain amino acids uses enzymes that are also involved in the citric acid cycle (Fig. 4). The MSBL1 SAGs lack the enzyme acetate kinase, which catalyses the transfer of phosphate from ATP to short chain aliphatic acids. However, genes for acetyl-CoA synthetase (which converts acetate to acetyl-CoA) were found in 16 of the SAGs. On the other hand, CO dehydrogenase/acetyl-CoA synthase (Table S2), which participates in the Wood-Ljungdahl pathway, through which CO_2_ is fixed under anaerobic conditions^24^, is present. The oxidative and reductive branches^25^ of this pathway are present in the MSBL1 indicating that both, one-carbon metabolism and carbon dioxide/carbon monoxide fixation might be possible. The occurrence of the various carbon fixation pathways is summarized in Fig. 4 and Supplementary Table S4. Among the autotrophic CO_2_ fixation pathways, the reductive acetyl-CoA pathway has the lowest energetic costs, requiring probably less than one ATP to produce pyruvate^26^. We cannot exclude the possibility that this pathway is used in the oxidative direction to oxidize acetate as energy substrate. This pathway has a requirement for metals, cofactors, strict anaerobic environment and substrates with low-reducing potential such as H_2_ or CO, which restricts the pathway to anoxic niches-such as the deep-sea brine pools. Many facultative autotrophic archaea often down-regulate the enzymes that are specifically required for CO_2_ fixation when organic substrates (such as acetate) are available^26^. Pyruvate formate lyase (present AAA259B11, AAA259E22 and AAAA259I09) catalyses the reversible conversion of pyruvate and coenzyme-A into formate and acetyl-CoA. Formate dehydrogenase detected in the SAGs may be involved in the oxidation of formate to CO_2_ and donating the electrons to NAD^+^ (since no cytochromes were detected). However, formate might also be reversibly incorporated into tetrahydrofolate by formyltetrahydrofolate ligase (although this is missing in the SAGs) and goes through a series of rearrangements resulting in the formation of 5-methyl-tetrahydrofolate (Fig. 4). The transfer of the methyl group of methyl-THF to carbon monoxide is mediated by a multi-enzyme complex catalysed by CO-methylating acetyl CoA synthase yielding acetyl-CoA^26^. Therefore, the acetyl-CoA decarboxylase/synthase complex (ACDS) bidirectionally links the tetrahydromethanopterin and tetrahydrofolate pathways with CO_2_ as the initial substrate (Fig. 4). Notably, the pterin-containing tetrahydromethanopterin and tetrahydrofolate serve as carriers of C_1_ fragments between formyl and methyl oxidation levels in both anabolic/ catabolic reactions^27^. Tetrahydromethanopterin may be involved in autotrophy (Wood-Ljungdhal pathway in some archaea) as well as purine biosynthesis whereas H4-folate could be used in the biosynthesis of methionine, serine and acetyl-CoA^28^. None of the core genes usually found in methanogenic archaea were detected in the MSBL1 SAGs (Supplementary Table S4).

Branched-chain amino acid transporters and permeases, transporters and genes for fatty acid beta-oxidation pathway were identified (Fig. 4, Supplementary Table S2). In the absence of fermentable sugars, these long-chain fatty acids (LCFA) could serve as an alternative carbon source for the MSBL1 group. The end product of LCFA biodegradation is acetyl-CoA, which can then be converted to pyruvate by 1.2.7.1, acetate by 2.8.3.1/6.2.1.13, acetoacetyl-CoA by 2.3.1.9, homocitrate by 2.3.3.14 or 3-carboxy-3-hydroxy-4-methylpentanoate by 2-isopropylmalate synthase (2.3.3.13).

### Sulphur metabolism

Sulphate, thiosulfate and sulfonates can be transported into the cell from the surrounding environment via ABC transporters and/or molybdate-tungstate transport system permeases or cysA/cysA2 proteins (Fig. 4, Supplementary Table S2). Assimilatory sulphate reduction occurs through ATP sulfurylase into adenylylsulfate (APS), which gets further reduced to either sulphite directly through the activity of adenylylsulfate reductase (1.8.99.2), or to form 3-phosphoadenylyl sulfate (PAPS) due to the activity of sulfate adenylyl transferase. Finally, PAPS is reduced to sulphite by PAPS reductase (Fig. 4). Therefore, in MSBL1, sulphate reduction is putatively assimilatory leading to synthesis of cysteine and homocysteine catalysed by cysteine synthase and cystathionine gamma-synthase, respectively (Supplementary Table S2). In six of the genomes, we detected a thiosulfate sulfurtransferase GlpE protein, which contains a rhodanese domain. Theoretically, the role of this protein is to transfer sulphur from thiosulfate to cyanide yielding sulphite and thiocyanate. The sulfoxide reductase catalytic subunit YedY protein, which was identified in AAA259E22 and AAA259I07, is an inner-membrane bound protein, which catalyses the reduction of sulfoxide to sulphite (Fig. 4). The sulphate reduction mechanism in MSBL1 probably proceeds in the same manner as in *A. fulgidus* and sulphate-reducing bacteria where by the CoB-CoM heterodisulfide reductase iron-sulphur subunit A protein transfers electrons via the adenosine 5′-phosphosulfate reductase (*Apr*AB 1.8.99.2 adenylylsulfate reductase subunit) from the reduced menaquinone pool in the membrane to activated sulphate (APS, adenosine-5′-phosphosulfate) forming sulphite. Localization prediction on the TMHMM server (http://www.cbs.dtu.dk/services/TMHMM-2.0/) shows that these reductases are located outside the membrane probably. The membrane-associated *dsr*MKJOP complex essential for sulphur oxidation as well as dissimilatory sulphite reductase are absent in MSBL1. Finally, five of the SAGs encode a gene identified as ferredoxin-nitrite reductase which is a homolog of the F_420_-dependent sulphite reductase^29^,^30^. It has been hypothesized that this enzyme may be involved in assimilatory nitrite/sulphite reduction^31^.

### Nitrogen metabolism

The SAG AAA259D14 encodes two genes *nrt*A and *nrt*D that are essential for nitrate uptake from the environment. Neither assimilatory nitrate reductases (Nas) nor respiratory nitrate reductase (Nar) were identified in the genomes. However genes encoding for a periplasmic nitrate reductase (napA) were identified in four genomes (Supplementary Table S2). It has been proposed that periplasmic nitrate reductase can participate indirectly in respiration as part of the electron transport chain when coupled to a proton-translocating enzyme, such as NADH dehydrogenase I (*Nuo*A-N enzyme), reviewed in references^32^,^33^. In SAG AAA259E22, the *nap*A gene is located on the same contig with heterodisulfide reductase (*hdr*ABC), tetrathionate reductase sub-unit B, Coenzyme F_420_ reducing hydrogenase and succinate dehydrogenase (iron sulfur and flavoproteins subunits). In genome AAA261O19, the gene is located on the same contig as NADH-quinone oxidoreductase (subunits ACDHIK). The link between nitrate reduction and electron transport is also supported by the occurrence of genes such for ferredoxin-nitrite reductase protein, cytochrome c-type protein *Nrf*B, 4Fe-4S ferredoxin iron-sulfur binding domain protein, electron transfer flavoprotein subunit alpha, electron transfer flavoprotein and periplasmic Fe-hydrogenase large subunit proteins (Supplementary Table S2). Other sources of nitrogen could be nitrile that is converted to ammonia catalysed by a nitrilase (3.5.5.1) or nitroalkene (also called nitro olefin) that is oxidized to nitrite by nitronate monooxygenase (EC: 1.13.12.16), whose orthologues were found in 15 genomes. Nitrilases act solely on carbon-nitrogen bonds to produce a carboxylate and ammonia. Eight SAGs encode an anaerobic nitric oxide reductase flavorubredoxin that can be used to detoxify nitric oxide using NADH^34^.

### Energy metabolism

Oxidative phosphorylation in MSBL1 consists of oxidoreductases, membrane bound hydrogenases and dehydrogenases, NADH-quinone/ubiquinone oxidoreductases, fumarate reductase and an ATPase complex (Supplementary Table S2). Based on the sub-unit composition of the NADH-quinone/ubiquinone oxidoreductase, the potential electron donor is NADH catalyzed by NADH dehydrogenase (found in 9 of the SAGs as shown in Table S2). The NADH dehydrogenase is a flavoprotein that contains iron-sulfur centres. Iron-sulphur binding proteins, oxidoreductases and fumarate reductase possibly contribute to energizing the cell membrane as well as general intracellular flow of electrons. Several genes encoding for coenzyme F420 hydrogenase and a putative hydrogenase maturation protease (EC 3.4.23.-) were identified in the SAGs (Supplementary Table S2). The CoB-CoM heterodisulfide reductase iron-sulfur protein (1.12.98.1) is similar to that of *Methanothermobacter fervidus* and is involved in sulphate reduction as discussed above. The subunit FrhB of F_420_-reducing hydrogenase carries the binding site for the prosthetic groups F_420_, FAD and a [4Fe-4S] cluster^35^. Putative K^+^-stimulated pyrophosphate-energized sodium pumps are probably involved in oxidative phosphorylation in the MSBL1.

### Transport

Transporters identified in the SAGs include ABC transport systems for branched-chain amino acid, arginine, ornithine, dipeptide, spermidine/putrescine, sugars and acids as well as for uptake of metal ions (Supplementary Table S2). These compounds provide the necessary substrates for numerous biosynthetic and degradation pathways. Additionally, ion transporters facilitate the flux of the different ions into, and also out of the cells (Fig. 4). For example, iron ions are essential for the synthesis of iron-sulphur clusters in the [NiFe] hydrogenases, formylmethanofuran dehydrogenases, heterodisulfide reductase, ferredoxins, and [Fe] hydrogenase. Phosphate is probably taken up by a *Pst*ABCS and *Pho*U system as described by Aguena and Spira^36^. This is confirmed by the occurrence of the respective genes/proteins involved in regulation and uptake of phosphorous from the environment. For example, in the SAG AAA259B11, genes for phosphate uptake regulation protein (PhoU), phosphate binding (PstS), ABC transporter permease protein (YqgH), phosphate Import ATP binding protein (PstB) and phosphate transport system permease protein PstA are all located on the same contig. Neither anion permeases nor sodium dependent phosphate transporters were identified in any of the SAGs. In microorganisms, molybdate ions are required for the synthesis of the molybdenum-dependent formylmethanofuran dehydrogenase, formate dehydrogenase and nitrogenases^37^,^38^. On the other hand, tungstate ions are required for the synthesis of the tungsten-dependent formylmethanofuran dehydrogenase^39^ and their uptake from the environment is mediated by a tungsten transport protein (WtpA).

### Stress response

Carbon starvation genes (carbon starvation-induced protein A) were detected in the SAGs AAA261C02 and AAA261O19, AAA833F18 and AAA833K04. This is a predicted membrane protein probably involved in peptide utilization when carbon becomes limiting^40^,^41^. Stress response genes include the small heat shock protein C4 and a universal stress protein YxiE (14 of the genomes). The universal stress protein UspA identified in four of the genomes is a small cytoplasmic bacterial protein whose expression is enhanced when the cell is exposed to stress agents^42^. Oxidative stress genes in MSBL1 include a putative oxidative stress-related rubrerythrin protein, putative superoxide reductases, glutaredoxins and thioredoxins. Glutathione and glutaredoxins are involved in disulphide reductions in the presence of NADPH and glutathione reductase^43^. Genes for resistance to heavy metals as well as antibiotics are listed in Supplementary Table S2. These include genes for the resistance to cadmium and arsenate as well as antibiotics such as danorubicin, methicillin, quinolones and tetracycline. The complete archaeal gene cluster for motility is missing in all the genomes though twitching motility protein PilT occurs in 21 SAGs. On the other hand, genes for pilus assembly are more widespread in the group (Supplementary Table S1) and could be responsible for secretion and cell-to-cell signalling.

## Discussion

MSBL1 have been presumed to be methanogens on the basis of phylogenetic placement or the presence of large amount of methane in the environments where they have been detected^3^,^9^,^10^,^44^,^45^. Phylogeny based on the Silva aligner places the MSBL1 within the class Thermoplasmata^16^ with identity scores between 83 to 86.9%. Previous phylogenetic placement of this group was summarized by Antunes *et al*.^5^. When shorter clone sequences are included in the analysis, the MSBL1 lie in the radiation of the uncultured Euryarchaeota group-SAGMEG^3^,^44^,^46^ or other uncultured groups^9^. However, the low bootstrap values in the phylogenetic trees do not allow for a clear placement at this point in time. In our analysis, we chose only full-length 16S sequences from the SAGs and comparative genomes from the NCBI database in order to have consistency between both the phylogenetic trees using 16S rRNA genes and core proteins. The MSBL1 group has been exclusively reported from hypersaline environments. The brine environment for example is one of the most extreme environments and therefore specifically adapted microorganisms probably have evolved mechanisms that enable them to adapt and thrive under these conditions. The common adaptation mechanisms have been previously described^11^,^47^. Ability of the MSBL1 archaea to import or synthesize osmolytes enables them to maintain intracellular osmotic balance and hence cope with salt stress in hypersaline environments where they have been reported. This is evidenced by the presence of transporters for glycine-betaine (Fig. 4) and also genes for biosynthesis of trehalose (Supplementary Figure S3). Furthermore, the slightly acidic proteome signature is associated with organisms employing the “salt out” strategy in contrast to the extreme halophiles that have a highly acidic proteome (Supplementary Figure S2) and use the “salt in” strategy^47^,^48^. MSBL1’s ability to operate between heterotrophy (sugar fermentation) and potentially autotrophic CO_2_ fixation (Fig. 2) highlights a possibility of a flexible mixotrophic lifestyle that might explain why MSBL1 is the major group reported for example in Lake Medee brine^4^ as well as in the metagenomic samples collected from the Atlantis II and Discovery brine pools^12^. Methanogenesis as we know it cannot occur in the absence of methyl-coenzyme M reductase as well as the associated cofactor (F_430_). We were not able to detect *mcr*A genes in the genomes nor were we able to amplify *mcr*A genes from the MDA-DNA that was used to generate the genome sequences. In addition, none of 15 core genes found in methanogenic archaea^49^ were detected in the MSBL1 SAGs (Supplementary Table S4). Moreover, at high salinity methanogenesis from H_2_ + CO_2_ or from acetate, dissimilatory sulphate reduction coupled to the oxidation of acetate, and autotrophic nitrification have been mentioned as some of the energy-producing reactions that are bioenergetically unfavourable^47^,^50^. Therefore, methane encountered in the brines could be from other biochemical processes or is produced by MSBL1 through a novel pathway independent of the canonical *mcr*A-associated pathway. For example, low amounts of methane observed in *Archaeoglobus*^51^ and in sulphate-reducing bacteria^52^ result from transfers of methyl groups by CO dehydrogenase. It is reported that the methyl group of N_5_-methyltetrahydromethanopterin can be reduced to methane and tetrahydromethanopterin by carbon monoxide (CO) dehydrogenase^53^,^54^. Assimilatory sulphate /nitrite reduction in the MSBL1 is catalysed by a ferredoxin-nitrite reductase^31^. Dissimilatory nitrate reduction serves to oxidize excess reducing equivalents^32^ potentially catalysed by the periplasmic nitrate reductase with electrons from formate dehydrogenase as the electron donor^55^. Stress response involves a repertoire of genes in the different SAGs (Supplementary Table S2). These include the universal stress protein UspA^42^, rubrerythrin (Rr) also found in anaerobic sulphate-reducing bacteria^56^ and rubredoxin^57^. Glutaredoxins and thioredoxins are proteins that act as antioxidants by facilitating the reduction of other proteins by cysteine thiol-disulphide exchange and therefore play a role in alleviating oxidative stress in MSBL1. The data presented here provide a first insight into the metabolism of this enigmatic uncultured archaeal lineage encountered in hypersaline environments. We are convinced that the metabolic reconstruction and genome sequences here will guide future isolation efforts.

## Materials and Methods

### Sampling sites and sample preparation

Samples used in this study were collected from the Atlantis II, Nereus, Erba and Discovery brine pools in the Red Sea (Table 2; Fig. 1) between 16^th^ and 29^th^ of November 2011 during the 3^rd^ KAUST Red Sea Expedition-Leg 2 on the vessel R/V *Aegaeo*.

Samples for the single-cell sorting were processed as follows: Small volumes of sample (*ca.* 30 ml) were collected and divided into two parts. The first aliquot of the sample was stored unfixed whereas the second part was fixed by adding glycerol (final concentration 10%) and immediately placed at –20 °C. Ten ml of the unfixed sample were transferred into a serum bottle and sent for cell sorting. Big volumes (ca. 480 L) of sample were collected into 20 L carboys, bubbled with nitrogen gas. Concentration was done using a Tangential Flow Filtration (TFF) system equipped with a 0.1μm cassette filter and coupled with a 5.0-μm pre-filter. A 10-ml portion of the concentrates was transferred to a serum bottle and sent for cell sorting. Cell sorting, lysis, whole genome amplification and SSU rRNA PCR were performed as described^58^ at the Bigelow Laboratory Single Cell Genomics Centre (http://www.bigelow.org). Amplification of the mcrA gene from the MDA reactions was done as published previously^59^.

### Sequencing, assembly, annotation

The SAG DNA was cleaned in preparation for sequencing using the ethanol/sodium acetate precipitation method and re-suspended in 25 μL of MilliQ water. Quantification of the DNA was performed using Quant-iT dsDNA HS assay kit and a Qubit fluorometer (Invitrogen GmbH, Karlsruhe, Germany) as recommended by the manufacturer. Sequencing was done at the Bioscience core facility, King Abdullah University of Science and Technology on an Illumina HiSeq 2000 platform. Assemblies of the single-cell amplified genomes (SAGs) were generated using a pipeline that employs assemblers designed for single-cell sequencing data including VelvetSC^60^, Spades^61^, and IDBA-UD^62^, along with several pre and post assembly data quality checks using Trimmomatic^63^. In our benchmarking tests, IDBA-UD showed better contig-level assemblies and the assemble contigs were used in further analysis. After quality control (described below), genome annotation for each of the SAGs was carried out as described in Alam *et al*.^64^. Briefly, given a set of DNA sequences from particular SAG, the Automatic Annotation of Microbial Genomes (AAMG) pipeline first detects rRNA and tRNA. To avoid prediction of Open Reading Frames (ORFs) in RNA detected regions, all DNA regions detected with RNA are masked, followed by ORF predictions using Prodigal^65^ and MetaGeneAnnotator^66^. After ORFs prediction is complete, a series of similarity searches are performed to select optimal gene annotations using UniProt, NCBI’s NR, NCBI’s Conserved Domain Database (CDD), KEGG database and finally Interproscan. All annotations, including DNA and ORF sequences are then stored in an integrative data-warehouse of microbial genomes (INDIGO, see methods in reference 64) for easy look up. As none of the SAGs represent complete genomes, the metabolic reconstruction in Fig. 4 represents our current working hypothesis for the metabolism of MSBL1.

### Quality control

Our approach towards decontaminating the draft assembly of SAGs was simple and some of the genomic features like GC content, size, gene content and tetranucleotide frequency (TNF)^67^ of the contigs were exploited. These filters were kept independent and a contig had to pass all filters in order to be put into the clean bin. A contig with %GC content lying outside + /− 10% range around the average was marked as potentially contaminated^68^. The calculation of the average G + C% for any draft assembly might be spurious and misleading if the assembly contains a lot of contaminations. We observed some real contigs (passing size, gene content and all filters) ending in the contamination bin just due to having slightly lower or higher G + C% around the range. This problem was overcome in two different ways. Firstly, if the analysis was done on a single SAG, the average GC content was calculated on the set of large contigs or the contigs constituting more than fifty per cent of the draft assembly (i.e N50 contigs). The second solution was applied when the analysis contained a group of SAGs belonging to same taxa and they needed to be cleaned in concert. In this case the single copy conserved genes were identified using Bio-Hal pipeline (PMID:21327165) for each of them and pooling their corresponding contigs to constitute a set (named “seed contigs”) on which G + C% was calculated. This single value of G + C content calculated on “seed contigs” could be applied for cleaning all SAGs of this group. Alternatively, the seed contigs could be separated for each SAG and G + C% calculated on different set. Size filter was relatively simple and cut-off could be fixed using the contig statistics of the draft assembly. For this cleaning the size threshold was 2000 bp i.e any contig below 2kb was discarded^68^. Nonetheless, a manual inspection of the contigs discarded just due to size filter is always advised. The smaller contigs (500 bp < x < 2000bp) might also contain some important gene. If majority (50% or more) of the genes in a scaffold/contig hits to the non-target phylum, that contig was discarded^68^. Binning of contigs was done at domain level either bacteria or archaea. Once the binning was complete based on the above three filters the clean bin and all bin were subjected to Canonical Correspondence Analysis (CCA) using TNF of the sequences and the contigs visualized on the plots^67^. Canonical Correspondence Analysis was done in R using Vegan package (https://cran.r-project.org/web/packages/vegan/index.html), while plotting was done in R using custom scripts developed in our group. The plot showing all contigs of the assembled genome gave a clear idea of the level of contamination in terms of phylogeny and G + C content profiles of contigs. The subsequent plot using only the clean contigs was much clearer and helped in finding out the few false positives, which were very dispersed but passed all the above three filters to be in clean bin. Manual inspection of such contigs was done to decide whether keep or discard them. The number of multiple single-copy conserved genes in the genome is a very important indicator of the contamination or might represent spurious assembly. To check the distribution of conserved cluster of orthologous groups (COGs) in the genome (to have an idea about “genome completeness”), we used different COG set for bacteria and archaea (adopted from Human Microbiome Project; R package Vegan was used (https://cran.r-project.org/web/packages/vegan/index.html). We observed in our single cell genomes data multiple copies of conserved genes could belong to multiple contigs. In most cases, the contig with largest size and more genes content was retained as part of the genome. Altogether, the QC pipeline takes care of the contamination present in the draft single cell genome using various genomic features both sequence-dependent and independent.

### Evolutionary relationships

The SAGs and the representative genomes were scanned for common marker genes (CMG) using the phylogenomic inference tool AMPHORA2^14^ along with its set of predefined marker genes. Identified marker genes were concatenated in the same order across all the samples and saved in multi-fasta format with headers being the sample names. The concatenated sequence in multi-fasta was then aligned using Muscle^69^ (reference) with default settings. Simple gblocks tool^70^-with default settings – was used to remove any ambiguous bases from the Muscle alignment. Phylogenetic trees were inferred from either trimmed alignments of nucleotide sequences (16S tree) or the amino-acid (protein tree) alignments of ten concatenated proteins present in all the genomes included in the analysis using the pthreads-parallelized RAxML^71^ version 7.2.8. The ten concatenated proteins are: translation initiation factor IF-2 (infB); 50S ribosomal proteins rpl18p, rpl19e, rpl32e, rpl5p, rpl6p, rpl7ae, and 30S ribosomal proteins rps28e, rps6e and rps8p. Fast bootstrapping was applied with subsequent search for the best tree^72^, the autoMRE bootstopping criterion^73^ and the LG model of amino acid evolution^74^ (with which these data yielded the highest log likelihood among all empirical protein models implemented in RAxML) in conjunction with gamma-distributed substitution rates^75^ and empirical amino acid frequencies. Tree searches under the maximum parsimony (MP) criterion were conducted with PAUP* version 4b10^76^ using 100 rounds of random sequence addition MP bootstrap support was calculated with PAUP* using 1000 replicates with ten rounds of heuristic search per replicate. The 16S rRNA gene datasets were analyzed in the same way, but using GTR as substitution model.

### *p*I estimation

In addition to the predicted protein-coding genes of our SAGs, we extracted the proteins-coding coding genes from GenBank files from the NCBI of exemplary extreme halophiles (*Halobacterium* sp. NRC1 and *Salinibacter ruber*), moderate halophiles (*Chromohalobacter salexigens, Idomarina loihiensis* L2TR, and *Nitrosococcus halophilus* Nc4), as well those from typical marine bacterioplankton (*Pelagibacter ubique* HTCC1062, *Pelagibacter* sp. HTCC 7211, *Nitrosopumilus maritimus* SCM1, and *Nitrococcus mobilis*). The isoelectric points (*pI*s) of these proteomes were calculated using the “iep” script in the EMBOSS software package (v6.5.1; http://emboss.sourceforge.net/what/) using the following settings: -amino 1 -termini YES –step 0.2.

### Data deposition

This Whole Genome Shotgun project has been deposited at DDBJ/EMBL/GenBank under the Bioproject PRJNA291812. The accession numbers for the Individual SAGS are LHXJ00000000-LHYO00000000.

## Additional Information

**How to cite this article**: Mwirichia, R. *et al*. Metabolic traits of an uncultured archaeal lineage -MSBL1- from brine pools of the Red Sea. *Sci. Rep.* **6**, 19181; doi: 10.1038/srep19181 (2016).

## Supplementary Material

Supplementary Information

Supplementary Table S1

Supplementary Table S2

Supplementary Table S3

Supplementary Table S4

## Figures and Tables

**Table 1:** Summary statistics of the clean contigs for SAGs described in this study.

SAG	Contigs	Length in bp	Longest	Smallest	Average	Genome Completeness (%)	
						104 Markers	191 Markers
**AAA261C02**	79	628,204	41,092	2,004	7,952	49.0	39.8
**AAA261F17**	62	527,268	47,307	2,094	8,504	32.7	21.5
**AAA261G05**	68	532,132	27,533	2,021	7,825	50.0	39.3
**AAA261O19**	99	731,948	41,274	2,022	7,393	41.3	35.6
**AAA261D19**	82	673,667	37,168	2,063	8,215	50.0	48.7
**AAA261F19**	82	655,981	44,778	2,016	7,999	49.0	45.5
**AAA259A05**	161	1,167,671	38,447	2,002	7,252	36.5	36.1
**AAA259D18**	56	387,594	17,623	2,039	6,921	14.4	6.8
**AAA259E17**	141	883,132	39,302	2,014	6,264	24.0	19.4
**AAA259E19**	211	1,424,127	37,031	2,003	6,749	42.3	37.2
**AAA259O05**	152	1,067,938	38,713	2,025	7,025	39.4	36.1
**AAA259B11**	118	828,207	32,654	2,022	7,018	19.2	23.0
**AAA259D14**	117	852,718	37,663	2,014	7,247	49.0	36.6
**AAA259E22**	100	825,701	37,800	2,026	8,257	14.4	16.8
**AAA259I07**	92	643,115	36,324	2,032	6,990	9.6	18.8
**AAA259I09**	215	1,350,271	42,722	2,011	6,280	56.7	54.5
**AAA259I14**	121	771,138	37,050	2,016	6,373	19.2	16.2
**AAA259J03**	148	896,033	45,149	2,003	6,054	29.8	31.4
**AAA259M10**	127	865,782	48,524	2,053	6,817	12.5	18.8
**AAA382A03**	88	428,827	17,372	2,040	4,866	14.4	16.8
**AAA382A13**	72	408,808	19,309	2,004	5,677	18.2	15.7
**AAA382A20**	111	925,397	41,226	2,010	8,336	36.5	27.7
**AAA382C18**	74	546,193	38,914	2,036	7,380	12.5	15.7
**AAA382F02**	34	336,176	29,731	2,226	9,887	25.9	34.0
**AAA382K21**	35	258,290	18,371	2,067	7,379	8.7	7.9
**AAA382M17**	63	378,040	25,329	2,007	6,000	32.7	22.5
**AAA382N08**	58	422,936	37,140	2,024	7,292	7.7	8.4
**AAA385D11**	35	306,358	26,504	2,012	8,753	8.7	12.6
**AAA385M02**	16	137,797	36,276	2,118	8,612	1.0	1.0
**AAA385M11** *	32	215,229	23,432	2,461	6,726	-	0.5
**AAA833F18**	24	269,215	63,868	2,373	11,246	10.6	9.9
**AAA833K04**	25	162,840	21,872	2,030	6,513	26.0	15.7

Contigs that were less than 2 kb were flagged as suspicious and omitted from the analysis. Genome completeness was computed using 104 and 191 conserved marker genes, respectively.

^*^None of the AMPHORA marker genes were detected in this genome.

**Table 2:** Metadata and geographical coordinates of sampled stations.

SAGID	Sampling Site	Layer	Temp. (°C)	Depth (M)	Salinity (%)	pH	Coordinates (La.t, N/ Long., E)
AAA833F18	Atlantis II Deep	Upper convective layer 1	≈ 54	2048	15.2	5.6	21°/38°
AAA833K04	Atlantis II Deep	Upper convective layer 1	≈ 54	2048	15.2	5.6	21°/38°
AAA259A05	Discovery	Brine	44.8	2141	26.2	6.2	21° 16.98′/38° 03.18′
AAA259B11	Discovery	Brine	44.8	2141	26.2	6.2	21° 16.98′/38° 03.18′
AAA259D14	Discovery	Brine	44.8	2141	26.2	6.2	21° 16.98′/38° 03.18′
AAA259D18	Discovery	Brine	44.8	2141	26.2	6.2	21° 16.98′/38° 03.18′
AAA259E17	Discovery	Brine	44.8	2141	26.2	6.2	21° 16.98′/38° 03.18′
AAA259E19	Discovery	Brine	44.8	2141	26.2	6.2	21° 16.98′/38° 03.18′
AAA259E22	Discovery	Brine	44.8	2141	26.2	6.2	21° 16.98′/38° 03.18′
AAA259I07	Discovery	Brine	44.8	2141	26.2	6.2	21° 16.98′/38° 03.18′
AAA259I09	Discovery	Brine	44.8	2141	26.2	6.2	21° 16.98′/38° 03.18′
AAA259I14	Discovery	Brine	44.8	2141	26.2	6.2	21° 16.98′/38° 03.18′
AAA259J03	Discovery	Brine	44.8	2141	26.2	6.2	21° 16.98′/38° 03.18′
AAA259M10	Discovery	Brine	44.8	2141	26.2	6.2	21° 16.98′/38° 03.18′
AAA259O05	Discovery	Brine	44.8	2141	26.2	6.2	21° 16.98′/38° 03.18′
AAA261C02	Atlantis II Deep	Brine -Interphase	57–63	2036	15.1–16.8	5.6	21°/38°
AAA261D19	Atlantis II Deep	Brine -Interphase	57–63	2036	15.1–16.8	5.6	21°/38°
AAA261F17	Atlantis II Deep	Brine -Interphase	57–63	2036	15.1–16.8	5.6	21°/38°
AAA261F19	Atlantis II Deep	Brine -Interphase	57–63	2036	15.1–16.8	5.6	21°/38°
AAA261G05	Atlantis II Deep	Brine -Interphase	57–63	2036	15.1–16.8	5.6	21°/38°
AAA261O19	Atlantis II Deep	Brine -Interphase	57–63	2036	15.1–16.8	5.6	21°/38°
AAA382A03	Nereus	Nereus Brine	30.1	2445	22.4	5.5	23° 11.53′/37° 25.09′
AAA382A13	Nereus	Nereus Brine	30.1	2445	22.4	5.5	23° 11.53′/37° 25.09′
AAA382A20	Nereus	Nereus Brine	30.1	2445	22.4	5.5	23° 11.53′/37° 25.09′
AAA382C18	Nereus	Nereus Brine	30.1	2445	22.4	5.5	23° 11.53′/37° 25.09′
AAA382F02	Nereus	Nereus Brine	30.1	2445	22.4	5.5	23° 11.53′/37° 25.09′
AAA382K21	Nereus	Nereus Brine	30.1	2445	22.4	5.5	23° 11.53′/37° 25.09′
AAA382M17	Nereus	Nereus Brine	30.1	2445	22.4	5.5	23° 11.53′/37° 25.09′
AAA382N08	Nereus	Nereus Brine	30.1	2445	22.4	5.5	23° 11.53′/37° 25.09′
AAA385D11	Erba	Erba Brine	28.2–28.5	2392	18.1	7.15	20° 43.80′/38° 10.98′
AAA385M02	Erba	Erba Brine	28.2–28.5	2392	18.1	7.15	20° 43.80′/38° 10.98′
AAA385M11	Erba	Erba Brine	28.2–28.5	2392	18.1	7.15	20° 43.80′/38° 10.98′

Physicochemical parameters recorded on site were depth, temperature, salinity and pH.
