# Supplementary material for: Metabolic traits of an uncultured archaeal lineage -MSBL1- from brine pools of the Red Sea
Source: Sci Rep. 2016 Jan 13;6:19181. doi: 10.1038/srep19181 (PMC4725937; doi:10.1038/srep19181)
Supplement: Supplementary Information [file srep19181-s1.docx]

**Metabolic traits of an uncultured archaeal lineage - MSBL1 - from brine pools of the Red Sea**

Romano Mwirichia^1^, Intikhab Alam^2^, Mamoon Rashid^1^, Vinu Manikandan^1^, Wail Ba-Alawi^2^, Allan Kamau^2^, David Kamanda Ngugi^1^, Markus Göker^3^, Hans-Peter Klenk^4^, Vladimir Bajic^2^ and *Ulrich Stingl^1^

**Supplementary Figure S1.** Maximum likelihood (ML) phylogenetic tree inferred from partial16S rRNA genes. In the tree are representatives of cultured archaea and MSBL1 sequences from the Red Sea, Mediterranean sea and Thetis II brines. The sequences were first aligned and then trimmed to a uniform length to cover the same sequence information. The branches are scaled in terms of the expected number of substitutions per site. Numbers above branches are support values from ML (left) and maximum parsimony (MP; right) bootstrapping. The tree was rooted with the bacteria present in the sampling

**Supplementary Figure S2:** Violin plots showing the distribution in the isoelectric point (*p*I) of the predicted proteomes of our MSBL1 SAGs (those with AAA*) relative to canonical extreme (in red) and moderate (in blue) halophiles, and typical marine bacterioplankton (in grey). The black dots denote the position the median pI, whereas the values on the right side of the panel are the analyzed total protein-coding genes. The red and blue vertical bars demarcate the median *p*I borders of the extreme halophiles (*p*I of 4.3–4.8; growing at >20% NaCl) and moderate halophiles (pI of 5.6– 6.8; growing at 5–20% NaCl; Koops et al., 1990; Kanekar et al., 2012), while the grey line shows a *p*I of 7.0. Abbreviations: ED-bsi, Erba Deep brine; AD-bsi, Atlantis II Deep brine-seawater interface; AD-ucl, Atlantis II Deep upper convenctive layer; DD-b, Discovery Deep brine; ND-b, Nereus Deep brine.


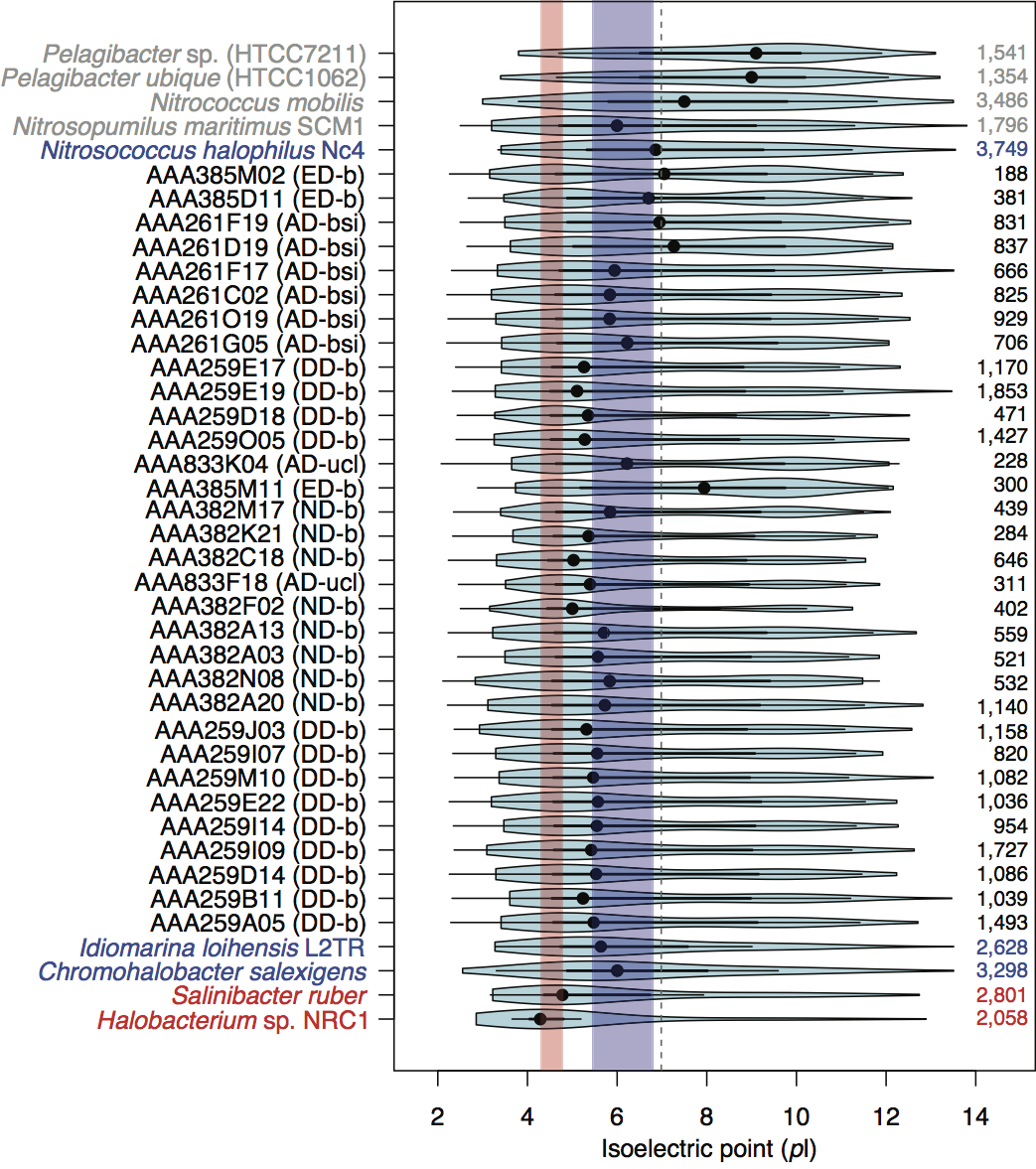


**Supplementary Figure S3: Predicted enzymes encoded in MSBL1 SAGs that are putatively involved in the metabolism and interconversion of polysaccharides:** (**1**) endoglucanase or aminopeptidase; (**2**) β-glucosidase A; (**3**) α-glucosidase; (**4**) α-glucanotransferase; (**5**) α-glucosidase, β-fructofuranosidase; (**6**) PTS system cellobiose-specific IIA component protein, PTS system (**7**) fructokinase; (**8**) glucose-6-phosphate isomerase; (**9**) phosphoglucomutase; (**10**) glucose-1-phosphate adenylyltransferase protein; (**11**) glycogen synthase protein; (**12**) MoeA region protein; (**13**) glycogen synthase (**14**) α-α-trehalose-phosphate synthase UDP-forming protein; (**15**) trehalose-phosphate phosphatase; (**16**) trehalose synthase protein; (**17**) alpha-amylase; (**18**) starch phosphorylase; (**19**) Trehalose synthase; (**20**) cellobiose phosphorylase. (**21**) α-1,4-glucanmaltose-1-phosphate maltosyltransferase; (**22**) trehalose-phosphate phosphatase.


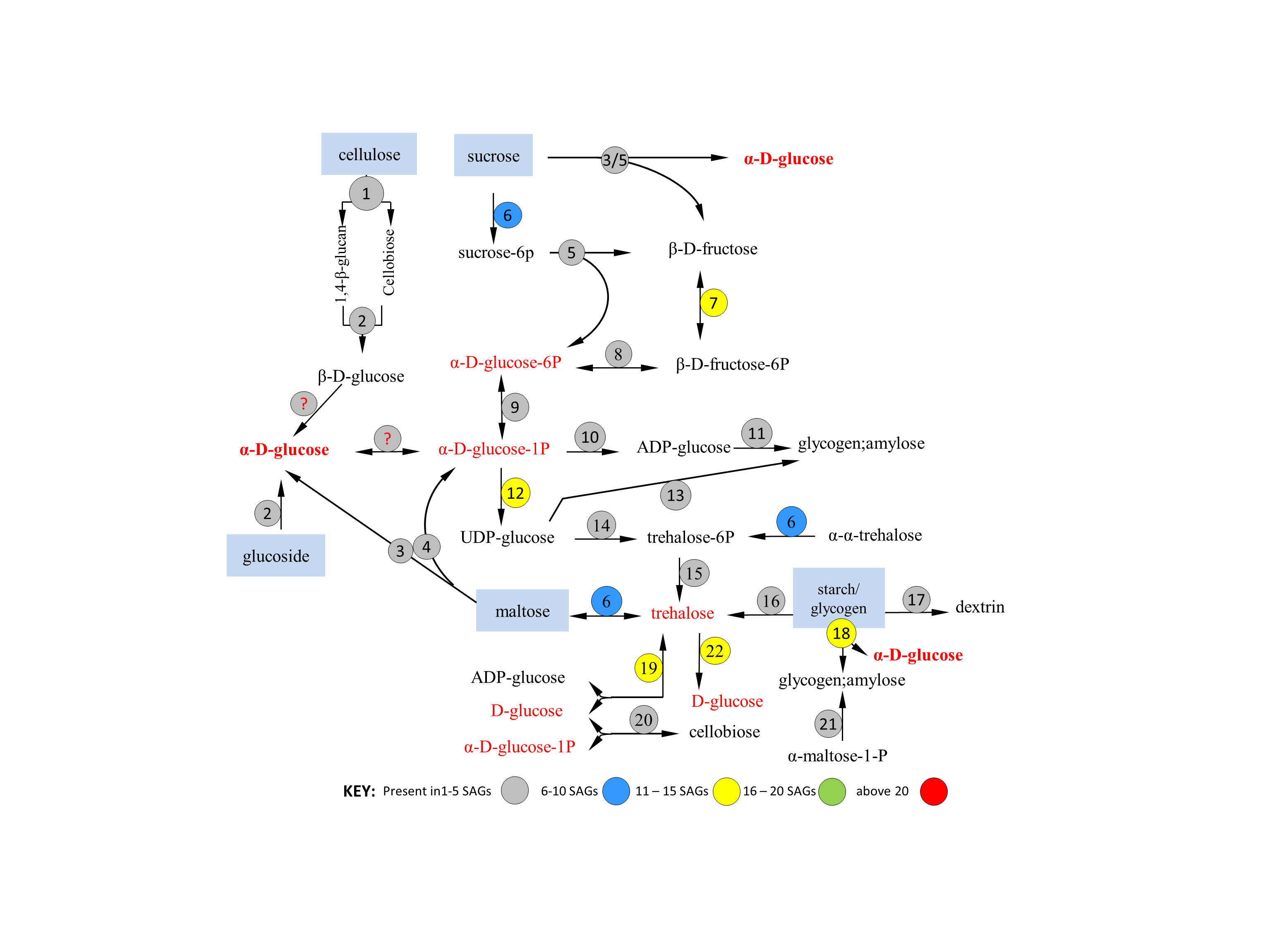


**References:**

Koops HP, Böttcher B, Möller UC, Pommerening-Roser A, Stehr G. (1990). Description of a new species of *Nitrosococcus*. Arch *Microbiol 154*: 244–248.

Kanekar PP, Kanekar SP, Kelkar AS, Dhakephalkar PK. (2012). Halophiles – taxonomy, diversity, physiology, and applications. In: Satyanaraya T, Johri BN, Prakash A (eds) Microorganisms in Environmental Management: Microbes and Environment. Springer: Dordrecht, pp 1–34.
